# Relationship between EGFR expression, copy number and mutation in lung adenocarcinomas

**DOI:** 10.1186/1471-2407-10-376

**Published:** 2010-07-19

**Authors:** Zhiyong Liang, Jing Zhang, Xuan Zeng, Jie Gao, Shafei Wu, Tonghua Liu

**Affiliations:** 1 Department of Pathology, Peking Union Medical College Hospital, Chinese Academy of Medical Science, Beijng 100730, China

## Abstract

**Background:**

This study was designed to investigate EGFR protein expression, EGFR copy number and EGFR mutations in lung adenocarcinomas, to explore the relationship of the three markers.

**Methods:**

EGFR status was analyzed in surgically resected lung adenocarcinoma samples from 133 Chinese patients by three methods: protein expression (n = 133) by standardized immunohistochemistry (IHC), gene copy number (n = 133) by fluorescence in situ hybridization (FISH), and mutation analysis using the Scorpion amplification refractory mutation system (ARMS) (n = 133).

**Results:**

The results showed that 68.4% of the samples were positive by IHC, 42.1% were positive by FISH, and 63.9% contained activating kinase domain mutations. EGFR mutations were more frequent in non-smoking patients (p = 0.008), and EGFR mutations were associated with EGFR FISH positivity (p < 0.0001). When using 10% positivity and 2+ as cutoffs, EGFR protein expression was significantly correlated with EGFR FISH positivity (p = 0.012) and EGFR mutations (p = 0.008) after Bonferroni correction.

**Conclusion:**

EGFR protein expression, EGFR copy number and EGFR mutations were closely related to each other. Standard methods and interpretation criteria need to be established.

## Background

Lung cancer is one of the leading causes of cancer-related deaths in the world. Recently, EGFR-targeted therapy has proven effective in treating non-small cell lung cancer (NSCLC). The epidermal growth factor receptor (EGFR, HER-1/ErbB1) is a receptor tyrosine kinase (TK) of the ErbB family, which consists of four closely related receptors: HER-1/ErbB1, HER-2/neu/ErbB2, HER-3/ErbB3, and HER-4/ErbB4. The agents approved for the treatment of NSCLC are monoclonal antibodies (MoAbs) directed against EGFR and small-molecule TK inhibitors (TKIs). Given the low response rate, the identification of the patients who are most likely to derive clinical benefit from EGFR-targeted therapy is important [1-6].

Increased EGFR gene copy number as detected by FISH was strongly correlated with response, progression-free survival (PFS) and overall survival (OS) after treatment with EGFR TK inhibitors (TKI) in previous studies. These results suggested that a high EGFR gene copy number is a strong indicator of TKI sensitivity [7,8].

Several clinical features were found to be associated with increased response rates to EGFR TKIs, including Asian ethnicity, non-smoking history, female gender and adenocarcinoma histology. EGFR mutations were reported to be associated with these clinical features in several clinical trials [9]. Mutations in the tyrosine kinase domain of EGFR were reported in the majority of tumors with dramatic responses to EGFR-targeted therapies, and an activating mutation of the EGFR tyrosine kinase domains was shown to be associated with EGFR TKI sensitivity [10-12]. EGFR gene mutations predicted increased overall survival of TKI-treated patients in some studies, but failed to indicate a survival benefit in other series of studies [10-14]. In recent studies, an association between EGFR mutations and high EGFR copy number was demonstrated [7,15].

It is still not clear whether EGFR protein expression could be a predictor of successful EGFR-targeted therapy. Due to the different antibodies, protocols and interpretation criteria used, as well as the different patient populations analyzed, EGFR protein expression in NSCLC has been variably reported. The association between EGFR protein expression as detected by immunohistochemistry (IHC) and the response to EGFR TKIs is controversial. The reported relationship between EGFR protein expression and EGFR copy number/EGFR mutation also varies in different studies [16,17].

Multiple methodological approaches have been used, including mutational analysis, fluorescence in situ hybridization, and immunohistochemistry. Conflicting results reflect the lack of standardization of the methodology and interpretation. In this study, we used the standardized PharmDx (Dako) IHC kit to analyze EGFR expression. We also analyzed gene copy number by FISH using the most standard probes (Vysis), and the mutations were analyzed by the stable and sensitive Scorpion amplification refractory mutation system (ARMS). We attempted to explore the relationship between EGFR protein expression, EGFR copy number, and EGFR mutation.

## Methods

### Patients

All of the specimens were selected by two pathologists, only patients with primary lung adenocarcinoma were selected, intrapulmonary metastases and recurrent disease were not included in this study. None of the selected patients were previously treated with chemotherapy, radiation or anti-EGFR therapy. Only cases with available EGFR immunohistochemistry, mutational status, and EGFR FISH data were analyzed. Clinical information included gender, age, smoking status, tumor stage and lymph node metastasis status. One hundred and thirty-three Chinese patients with lung adenocarcinomas were selected from 886 lung cancer patients who underwent surgery at the Department of Surgery, Peking Union Medical College Hospital from Jan. 2000 to Jan. 2008. The patient group consisted of 62 males and 71 females, with an average age of 60 years. Cancer staging was classified according to the TNM cancer staging system of the American Joint Committee of Cancer (13): stage I, 69 cases; stage II, 17 cases; stage III, 33 cases and stage IV, 14 cases. The World Health Organization Classification of Tumors was used for histological classification and grading (18). The institutional review board at the Peking Union Medical College Hospital approved this study, and informed consent was obtained from all patients.

### Sample preparation

All specimens were fixed in 10% buffered formalin and embedded in paraffin according to standard procedures. All the tissues were fixed immediately after surgical resection, time from tissue acquisition to fixation was as short as possible; samples were fixed in 10% neutral buffered formalin (avoiding Bouin or any fixative containing heavy metal ions) for 6-48 hours; samples were sliced properly after appropriate gross inspection and margins designation and placed in sufficient volume of 10% neutral buffered formalin.

Serial sections (4-5 μm thickness) placed on positively charged slides (MENZEL-GLASSER, GERMAN) were used for hematoxylin and eosin staining, immunohistochemistry, and FISH detection of EGFR.

### FISH analysis of EGFR copy number

EGFR FISH analysis was carried out using the LSI EGFR SpectrumOrange/CEP 7 SpectrumGreen probe (Vysis, Abbott Laboratories) according to the manufacturer's protocol. Sections were incubated at 56°C overnight, deparaffinized by washing in CitriSolv (Fisher Scientific, Pittsburgh, PA), and dehydrated in 100% ethanol. After incubation in 2× saline sodium citrate buffer (2× SSC, pH 7.0) at 75°C for 15-25 minutes, sections were digested with proteinase K (0.25 mg/mL in 2× SSC; pH 7.0) at 37°C for 15-25 minutes, rinsed in 2× SSC at room temperature for 5 minutes, and dehydrated in a series of increasing concentrations of ethanol (70%, 85%, and 100%). The EGFR/CEP 7 probe set was applied to the selected area based on the presence of tumor foci on each slide, and the hybridization area was covered with a glass coverslip and sealed with nail polish. The slides were incubated at 80°C for 8-10 minutes for co-denaturation of the chromosomal and probe DNA and were then hybridized at 37°C for 20-24 hours. Post-hybridization washes were performed in 1.5 M urea and 0.1× SSC (pH 7.0 - 7.5) at 45°C for 30 minutes, and in 2× SSC for 2 minutes at room temperature. After the samples were dehydrated in ethanol as above, 4', 6'-diamidino-2-phenylindole (DAPI) in phosphate-buffered saline and glycerol (Vysis) was applied for chromatin counterstaining. FISH analyses were performed independently by two authors who were blinded to the clinical characteristics of the patients and to all other molecular variables. For EGFR FISH analyses, 60 nuclei were scored for signals from both DNA probes using an Olympus BX51TRF microscope (Olympus, Japan) equipped with a triple-pass filter (DAPI/Green/Orange; Vysis) at a final magnification of 1000×.

Chromosome 7 polysomy and monosomy were defined as the presence of ≥ three signals and one signal, respectively, in more than 20% of the tumor cells. EGFR gene status was classified into six categories according to the frequency of tumor cells with specific copy numbers of the EGFR gene and the chromosome 7 centromere as described elsewhere [7]: disomy (≤ 2 copies in ≥ 90% of cells), low trisomy (≤ 2 copies in ≥ 40% of cells, 3 copies in 10 - 40% of cells, and ≥ 4 copies in < 10% of cells), high trisomy (≤ 2 copies in ≥ 40% of cells, 3 copies in ≥ 40% of cells, and ≥ 4 copies in < 10% of cells), low polysomy (≥ 4 copies in 10 - 40% of cells), high polysomy (≥ 4 copies in ≥ 40% of cells), and gene amplification (a: presence of tight EGFR gene clusters(≥4 spots) in ≥10% tumor cells;b: a ratio of the EGFR gene to chromosome 7 of ≥ 2, c: ≥ 15 copies of EGFR per cell in ≥ 10% of cells). Based on the EGFR gene status, patients were further classified into two groups: 1) EGFR FISH-negative or low gene copy (disomy, low trisomy, high trisomy, and low polysomy) and 2) EGFR FISH-positive or high gene copy (high polysomy and gene amplification)[18]. For each FISH preparation, known positive and negative cells were used as controls.

### Scorpion ARMS analysis of EGFR mutations

The tumor specimens were fixed with 10% neutral buffered formalin and embedded in paraffin. Using a NucleoSpin tissue isolation kit (Machery-Nagel, Germany), DNA was extracted from tumor tissues derived by manual microdissection carried out to enrich tumor cells. EGFR mutations of exon 18-21 were detected using the DxS ARMS EGFR29 mutation test kit (DxS Limited, Manchester, UK). Twenty-nine of the most common somatic mutations of the EGFR gene can be detected by the kit.

### Immunohistochemical analysis of EGFR expression

Immunohistochemistry for EGFR was performed using the EGFR pharmDx kit (DakoCytomation, Denmark) according to the manufacturer's instructions. Antibody binding was visualized using the EnVison detection kit (DakoCytomation, Denmark).

Immunohistochemical staining for EGFR was scored as follows: 0, no discernible staining or presence of background staining only; 1+, equivocal discontinuous membrane staining; 2+, unequivocal membrane staining with moderate intensity; and 3+, strong and complete plasma membrane staining. More than 10% of the cells were required to meet the criteria for EGFR analysis. Samples with more than 10% of the tumor cells showing membranous (partial or complete) staining at the 2+ and 3+ staining levels were considered to be EGFR IHC-positive, and the highest score obtained among different areas of the same tumor was used as the final EGFR IHC result for that tumor. To determine which criteria were more suitable, two other evaluation criteria were used in this study. The first was that samples with more than 10% of tumor cells showing membranous (partial or complete) staining of any intensity were considered to be EGFR IHC-positive. The second criterion we used was the semiquantitation score defined by Capuzzo et al. [7], with the modification that we evaluated membranous staining and determined four levels of intensity (0, 1+, 2+ and 3+) according to the instructions of the manufacturer (Dako). The IHC score was calculated by multiplying the staining intensity and the fraction of positive cells (0-100%). The scores were 0-300 in different tumors, and scores of more than 100 were considered as indicative of EGFR IHC positivity [19,20].

### Statistical analysis

Statistical analyses were performed using Pearson's Chi-squared test or Fisher's exact test to determine significant clinicopathological differences between EGFR expression in positive and negative tumors, between EGFR FISH-positive and FISH-negative tumors, and between tumors with and without EGFR mutations. These tests were also used to determine the association between EGFR protein expression, EGFR FISH results, and EGFR mutations. Bonferroni correction was performed to adjust for multiple comparisons, differences with P < 0.05/comparison times were considered significant.

## Results

### EGFR copy number in lung adenocarcinomas

Eleven (8.3%) of the 133 cases showed EGFR amplification, 45 cases (33.8%) showed high polysomy, 38 cases (28.6%) showed low polysomy, 1 (0.7%) case showed high trisomy, 15 cases (11.3%) showed low trisomy, and 23 cases (17.3%) showed disomy. Fifty-six cases (42.1%) showed FISH positivity according to the criteria of Capuzzo et al[7]. After Bonferroni correction for 5 comparisons, P < 0.01 were considered significant, EGFR FISH positivity was not associated with gender, smoking status, age, lymph node metastasis or tumor stage (P ≥ 0.01) (Table 1).

**Table 1:** Relationship between EGFR mutation, EGFR copy number and clinicopathological characteristics of lung adenocarcinoma

	IHC			Mutation			FISH		
						
	+	-	P value	+	-	P value	+	-	P value
Gender									
Male	41	21	0.595	34	28	0.042	27	35	0.753
Female	50	21		51	20		29	42	
Age									
>60	50	23	0.984	51	22	0.115	30	43	0.795
<60	41	19		34	26		26	34	
Smoking status									
Positive	61	25	0.400	62	24	0.008	36	20	0.938
Negative	30	17		23	24		50	27	
Lymph node metastasis									
Positive	59	28	0.836	58	29	0.363	33	54	0.180
Negative	32	14		27	19		23	23	
Stage									
I	47	22	0.937	45	24	0.744	22	47	0.021
II	15	2		12	5		11	6	
III	21	12		19	14		14	19	
IV	8	6		9	5		8	6	

After Bonferroni correction, P < 0.01 was considered as significant, only the association between EGFR mutation and smoking status was statistically significant.

### EGFR mutations in lung adenocarcinomas

Eighty-five (63.9%) of the 133 cases showed EGFR mutations, which included 2 exon 18 G719X mutations (one also had an exon 20 S768I mutation), 39 exon 19 deletions, 4 exon 20 insertion mutations, 3 exon 20 S768I mutations (one also had an exon 18 G719X mutation), 35 exon 21 L858R mutations (one also had an exon 20 T790 M mutations), and 3 exon 21 L861Q mutation. After Bonferroni correction for 5 comparisons, P < 0.01 were considered significant, EGFR mutations were significantly associated with smoking status (non-smoking vs. smoking, p = 0.008), and were not associated with age, gender, lymph node metastasis or tumor stage (p ≥ 0.01) (Table 1).

### Association between EGFR copy number and EGFR mutations

In our results, EGFR FISH positivity was significantly associated with EGFR mutations (p < 0.001). Forty-five of the 56 (80.4%) FISH-positive cases, which included 7 amplifications and 38 instances of high polysomy showed EGFR mutations. Forty of the 77 (52%) FISH-negative cases, which included 23 low polysomy cases, 10 low trisomy cases, and 7 disomy cases showed EGFR mutations. There were no significant differences in the EGFR FISH-positive rate among different mutation patterns (p ≥ 0.05) (Table 2).

**Table 2:** Relationship between EGFR copy number and mutation

Mutation	FISH	
	
	Positive	Negative
Positive	45	40
negative	11	37

P value	P < 0.0001	

### EGFR protein expression in lung adenocarcinomas

Sixty-one (45.8%) of the 133 cases showed EGFR expression levels corresponding to 3+, 30 (22.6%) cases showed EGFR levels of 2+, and 23 (17.3%) cases showed EGFR levels of 1+; the remaining 19 (14.3%) cases showed level 0 staining (n = 133 lung adenocarcinoma samples).

### Correlation of EGFR expression, EGFR copy number and EGFR mutations

According to the criteria that samples with more than 10% of tumor cells showing membranous staining of any intensity should be considered as EGFR IHC-positive, 114 cases were IHC-positive and 19 cases were IHC-negative. After Bonferroni correction for 2 comparisons, P < 0.025 were considered significant, the FISH-positive rate was not significantly different between IHC-positive and IHC-negative groups (p = 0.132), and the mutation rate was not significantly different between the IHC-positive and IHC-negative groups (p = 0.105) (Table 3).

**Table 3:** Relationship between EGFR protein expression and EGFR copy number, and mutation

IHC criteria	Mutation			FISH		
				
	positive	negative	P value	positive	negative	P value
First criteria						
Positive (>10% tumor cells, ++ and +++)	65	26	0.008	45	46	0.012
Negative	20	22		11	31	
						
Second criteria						
Positive (>10% tumor cells, any intensity)	76	38	0.105	51	63	0.132
Negative	9	10		5	14	
						
Third criteria Positive (H Score ≥ 100)	62	24	0.008	42	44	0.027
Negative(H Score < 100)	23	24		14	33	

After Bonferroni correction, P < 0.025 was considered as significant. For using the first IHC criteria, IHC were significantly associated with mutation and FISH. For using the second IHC criteria, no significant association was found between IHC and mutation or FISH. For using the third IHC criteria, IHC was significantly associated with mutation, approached significant associated with FISH(P = 0.027,close to 0.025)

**Figure 1:**
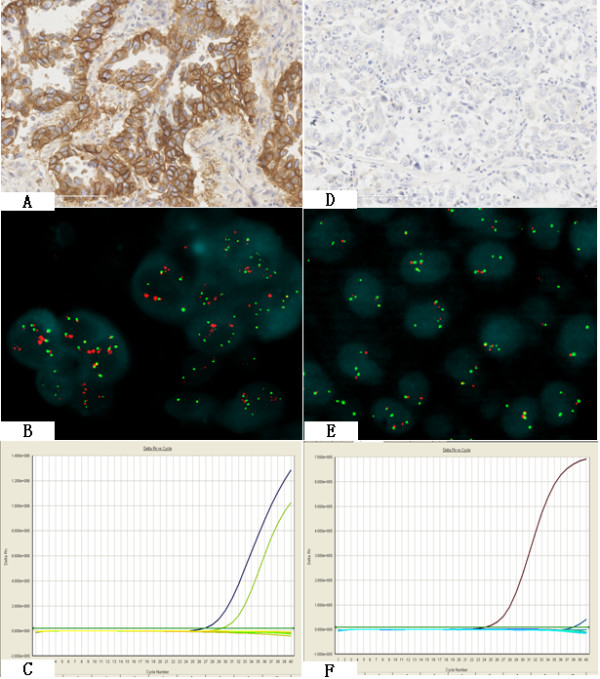
**The consistency of three biomarkers**. A-C: Case 1, three biomarkers were all positive in the same case, A: IHC positive(3+), B: FISH positive (amplification), C: Curves for exon 19 using the Scorpions ARMS method, if only one ascending curve showed the wild-type, more than two ascending curves showed the mutation. Two ascending curves in this case indicated mutation(the left curve represented wild type, the right curve represented mutant, exon 19 deletion). D-F: Case 2, three biomarkers were all negative in the same case. D: IHC negative, E: FISH negative(low trisomy), F: mutation negative(one ascending curve indicated EGFR wild type).

**Figure 2:**
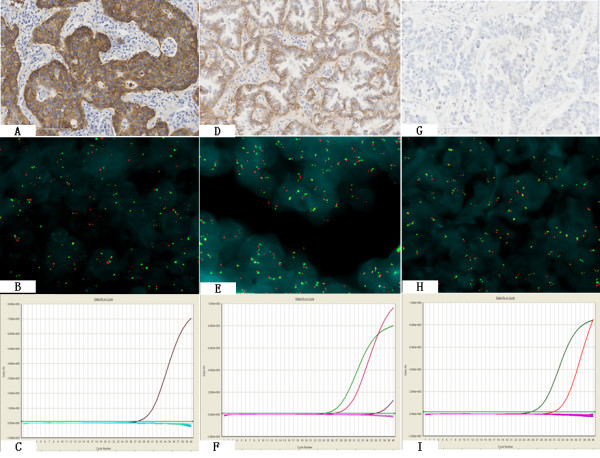
**The inconsistency of three biomarkers**. The three biomarkers were not completely overlap, some IHC positive or FISH positive patients were mutation negative. A-C: Case 3, A: IHC positive(3+), B: FISH positive(amplification),C: mutation negative(one ascending curve indicated EGFR wild type). D-F: Case 4, D: IHC positive(2+), E: FISH negative(disomy), F: Curves for exon 19 using the Scorpions ARMS method, if only one ascending curve showed the wild-type, more than two ascending curves showed the mutation. Two curves in this case indicated mutation(the left curve represented wild type, the right curve represented mutant, exon 19 deletion). G-I: Case 5, G: IHC negative, H: FISH positive(high polysomy), I: Curves for exon 21 using the Scorpions ARMS method. Two ascending curves in this case indicated mutation (the left curve represented wild type, the right curve represented mutant, exon 21 L858R mutation)

According to the criterion that samples with more than 10% of tumor cells showing membranous staining at levels 2+ and 3+ should be considered to be EGFR IHC-positive, 91 cases were IHC-positive and 42 cases were negative. After Bonferroni correction for 2 comparisons, P < 0.025 were considered significant, the FISH-positive rate was significantly higher in the IHC-positive group than in the IHC-negative group (p = 0.012), and the mutation rate was significantly higher in the IHC-positive group than in the IHC-negative group (p = 0.008) (Figure 1, Table 3). But the patients positive for the three EGFR markers did not completely overlap; some of FISH-positive and mutant samples was IHC-negative (Figure 2).

According to semiquantitation criteria, 88 cases were IHC-positive and 46 cases were negative. After Bonferroni correction for 2 comparisons, P < 0.025 were considered significant, the mutation rate was significantly higher in the IHC-positive group than in the IHC-negative group (p = 0.008), the FISH-positive rate was higher in the IHC-positive group than in the IHC-negative group (approached significance, p = 0.027) (Table 3).

Similar results were obtained using either of the latter two criteria. The results indicated that EGFR IHC positivity was significantly correlated with EGFR mutations and EGFR FISH positivity (the association between the third EGFR IHC criteria and EGFR FISH positivity was close to statistically significant). Due to its easier application, we used the criterion that samples with more than 10% of tumor cells showing membranous staining at levels 2+ and 3+ should be considered to be EGFR IHC-positive in our following analysis. There were no significant differences in the rates of IHC positivity in patients of different age, gender, smoking status, lymph node metastasis and stage (p ≥ 0.01 after Bonferroni correction).

## Discussion

Due to the potential high benefit of EGFR TKI therapy for treatment of lung adenocarcinomas, several clinically trials have focused on lung adenocarcinomas. Because a low proportion (<30%) of patients respond to EGFR TKI, patient selection is very important for initiating EGFR TKI therapy [1-6]. EGFR gene copy number determined by FISH, protein expression determined by IHC and EGFR tyrosine kinase mutations are all potential markers to be used as selection criteria in EGFR-targeted therapy. Multiple methodological approaches have been used in the detection of EGFR mutations, EGFR copy number and EGFR protein expression, but the results were contradictory. It is important to standardize the approach and decide which assays are best to predict patient responses to targeted therapies.

It was demonstrated that mutations in exons 18 to 21 of the tyrosine kinase domain of EGFR were correlated with a high response to EGFR TKIs in lung adenocarcinomas. In previous studies, EGFR mutations were reported in about 22% to 67%, 10% to 24%, and 3% to 25% of Asian population, Southern European, and American populations with lung adenocarcinomas [10,12,14,15,21-26]. In this study, 63.9% (85/133) of patients with lung adenocarcinomas had EGFR mutations. The higher frequency of mutations can most likely be attributed to the sampling from a Chinese population and the sensitive Scorpion ARMS method used. Although direct sequencing is still the gold standard method for mutation screening, many more sensitive methods have been developed, and Scorpion ARMS has been used in several clinical trials. Scorpion ARMS can detect a lower proportion of mutant alleles than direct sequencing; we demonstrated that Scorpion ARMS was more sensitive than direct sequencing in detecting EGFR mutations in lung carcinomas in our previous study [27]. Others reported that some cases with mutations are missed by direct sequencing of clinical tumor samples; however, these cases could be detected by more sensitive techniques. In this study, about 90% (74/85) of EGFR mutations consisted of deletions in exon 19 and L858R mutations in exon 21, and EGFR mutations were significantly associated with smoking status, consistent with previous reports, but not associated with gender, this may be caused by small sample size in our group. Other less common mutations were also identified in our study, including one T790 M mutation that has been demonstrated to result in EGFR TKI resistance in clinical trials.

High EGFR gene copy number/amplification has been reported in 7% to 45% of lung carcinomas. This large range may be due to variations in techniques, criteria for determining positivity, and interobserver variability [7,8,15,20,28]. In our study, standard Vysis EGFR FISH probes and the Capuzzo et al. criteria were used to evaluate EGFR copy number in lung adenocarcinomas. We found that 42.1% (56/133) of lung adenocarcinomas showed EGFR FISH positivity, and that EGFR FISH positivity was more frequent in late stages than in early stages of lung adenocarcinomas (although no statistically significant difference showed after Bonferroni correction, but there was trend show difference, probably due to the reason of low volume samples.). Awaya et al. [29] detected FISH only in the invasive components of adenocarcinomas, and Kim et al. [30] frequently observed increased EGFR in advanced stages of lung adenocarcinoma. This may be consistent with previous reports of poor prognosis of lung carcinomas with higher EGFR copy numbers. Our studies and several others from Western and American populations demonstrated that high EGFR copy numbers were significantly associated with EGFR mutations, contrary to the result published by Sasaki et al. [31] in a study of Japanese patients in which no correlation was found between EGFR copy number and EGFR mutation. There may be reasons other than ethnicity for this discrepancy. The relationship between high EGFR copy number and EGFR mutation is complex. Recent studies report a mutant allele specific imbalance of oncogenes in tumor cells harboring gene mutation; copy number gain of EGFR usually occurred in the cells with an EGFR mutation[32]. Previous studies reported that EGFR mutant alleles were amplified selectively, resulting in a high EGFR copy number[15]. Yatabe, et al. [33] reported that EGFR amplification was acquired during invasive growth of lung adenocarcinoma with EGFR mutations. Soh, et al. [34] have found that an increase in EGFR copy number was a relatively late event in NSCLC pathogenesis and that EGFR mutation preceded EGFR amplification. These may partially explain the association between high EGFR copy number and EGFR mutation, the precise mechanism needs to be clarified in future studies.

EGFR expression has been variably reported in 27 to 83% of NSCLC cases, and there are conflicting results on the prognostic implication of EGFR expression in NSCLC.

Different EGFR IHC results highly depend on the type of antibody, procedure protocols, selection of scoring methods, and cut-offs implemented. A standard method has not been adopted, and the significance of EGFR protein expression in NSCLC remains contradictory [35-39]. To select the best evaluation standard for EGFR IHC, we compared three different criteria in our study. When we used the criterion that samples with more than 10% of tumor cells showing membranous staining of levels of 2+ and 3+ should be considered to be EGFR IHC-positive, the results were statistically similar to those obtained using the Capuzzo et al[7] criteria. EGFR protein expression was significantly associated with EGFR copy number and EGFR mutations, and this was consistent with results from most of the previous studies[40]. In contrast, if we used 10% positivity regardless of intensity as a cut-off, the results were quite different, and no correlation was found between EGFR protein expression, EGFR copy number and EGFR gene mutation.

These results suggest that the establishment of standard methods and interpretation of standards is very important in clinical practice.

We prefer to use 10% positivity and an intensity level of 2+ as cut-off criteria. In our study, 68.4% (91/133) of lung adenocarcinomas were EGFR IHC-positive according to our interpreting criteria. EGFR protein expression was significantly associated with EGFR copy number and EGFR mutation, but not associated with gender, age, smoking status, lymph node metastasis or stage.

The results suggest that EGFR protein expression may predict EGFR gene status (including copy number and mutation) to some extent. However, the patients positive for the three EGFR markers did not completely overlap; a proportion of FISH-positive and mutant samples was IHC-negative. Thus, the mechanism needs to be clarified in the future.

## Conclusion

EGFR protein expression, EGFR copy number and mutations were investigated in this study. Our results revealed that the deletion in exon 19 and the L858R point mutation were the major EGFR mutations in lung adenocarcinomas, and EGFR mutations were significantly associated with smoking status. Furthermore, EGFR copy number was significantly associated with EGFR mutation, and EGFR protein expression was significantly correlated with EGFR copy number and mutation. Standard methods and criteria should be established for patient selection for EGFR target therapy. Further studies will be required to determine whether EGFR copy number and EGFR protein expression analysis are suitable for individualized EGFR targeted therapy.

## Competing interests

The authors declare that they have no competing interests.

## Authors' contributions

ZYL designed the experiment and drafted the manuscript, JZ participated in specimen collection and data analysis XZ participated in experiment design and performed FISH analysis, JG performed immunohistochemistry staining and mutation tests, SW carried out FISH analysis, THL conceived of the study, and participated in its design and coordination. All authors read and approved the final manuscript.

## Pre-publication history

The pre-publication history for this paper can be accessed here:

http://www.biomedcentral.com/1471-2407/10/376/prepub
